# The Tumor Microenvironment and Immunotherapy of Oropharyngeal Squamous Cell Carcinoma

**DOI:** 10.3389/fonc.2020.545385

**Published:** 2020-12-21

**Authors:** Marij J. P. Welters, Saskia J. Santegoets, Sjoerd H. van der Burg

**Affiliations:** Medical Oncology, Oncode Institute, Leiden University Medical Center, Leiden, Netherlands

**Keywords:** tumor microenvironment, immunotherapy, oropharyngeal cancer, T cells, myeloid cells, clinical outcome, survival

## Abstract

Oropharyngeal squamous cell carcinoma (OPSCC) develops as a consequence of several mutations in the tumor suppressor pathways or after a progressive infection with high risk human papillomavirus (HPV). The dismal side effects of the current standard of care and the clear involvement of the immune system has led to a surge in clinical trials that aim to reinforce the tumor-specific immune response as a new treatment option. In this review, we have focused on the most recent literature to discuss the new findings and insights on the role of different immune cells in the context of OPSCC and its etiology. We then applied this knowledge to describe potential biomarkers and analyzed the rationale and outcomes of earlier and ongoing immunotherapy trials. Finally, we describe new developments that are still at the preclinical phase and provide an outlook on what the near future may bring, now that several new and exciting techniques to study the immune system at the single cell level are being exploited.

## Introduction

Head and neck cancer is the sixth most prevalent cancer type and mainly consists of squamous cell carcinoma [90%; HNSCC (1, 2)]. The tumor can develop in the oral cavity, the larynx, pharynx (hypopharynx, nasopharynx, or oropharynx) and in the sinonasal tract. While the incidence of head and neck cancer located at most oral sites is marginally decreasing due to the knowledge of tobacco and alcohol as risk factors for its development, the incidence of oropharyngeal carcinomas is increasing, especially in the developed world (3). Oropharyngeal squamous cell carcinomas (OPSCC) include oropharyngeal, tonsillar and base-of-tongue tumors. A high percentage (60–80%) of the OPSCC are induced by high risk human papillomavirus type 16 (HPV16) (4–6). Patients with HPV+ OPSCC often tend to be younger, approximately 75% is male, a minority smokes and they often have lymph node metastasis when first visiting the clinic (7, 8). HPV is a double stranded DNA virus encoding for early and late (envelop) proteins (9, 10). The early proteins E6 and E7 are oncoproteins and responsible for the malignant transformation of HPV infected epithelial cells and maintenance hereof (11, 12). Patients with HPV16+ OPSCC display a longer overall survival (OS) and a lower recurrence rate after standard of care treatment than patients with HPV-negative OPSCC (13, 14).

Only recently the difference between these two OPSCC entities has been acknowledged and a debate on treatment has been started (15, 16). To understand the differences between the HPV+ and HPV-negative tumors, in-dept analysis have been performed on various levels including genetics (DNA), epigenetics, (micro)RNA and the immune system (7). De-intensified treatment, by lowering the dose of the (chemotherapeutic) drug or radiotherapy or by replacement of the drug, have been suggested in order to decrease side effects particularly in patients with HPV+ OPSCC (15, 16). This led to inferior survival and new treatment designs are required (17). Immunotherapy may form a new effective treatment in OPSCC and is currently under investigation. While it is clear that during HPV infection and subsequent transformation several ways are exploited to escape from the immune system (18–22), HPV16 E6/E7-specific T-cells are often detected in OPSCC tumors and their presence is associated with improved clinical outcome (6, 23, 24). In addition, a whole series of articles exists on the association between intratumoral T-cell infiltration and better clinical outcome after standard of care therapy (25–28). These type of data paved the way for immunotherapeutic strategies to harness the immune response to OPSCC. Indeed, blockade of CTLA-4 and/or PD-1 has been studied and showed a survival benefit in a subset of patients with different types of HNSCC when compared to the standard of care treatment arm (29). The vast majority of patients did not benefit from this treatment, illustrating the requirement for in-depth studies on systemic and local host-tumor interactions. In particular, studies on the tumor immune microenvironment (TME) are a prerequisite to understand what hurdles are at play and need to be overcome in order for the immune system to effectively control tumor growth.

A search in PubMed using the key terms “Oropharyngeal cancer immunity”, “Oropharyngeal cancer myeloid cells”, “Oropharyngeal cancer immunotherapy trial”, and “Head and Neck squamous cell carcinoma immunotherapy trial” was performed for articles published in the last 10 years (until august 2020) describing cohorts of patients with head and neck cancer, of which at least 25% had OPSCC and the results were not typical for one type of HNSCC, or with <25% of OPSCC patients but with results specific for the OPSCC group. Also, studies comparing the results between HPV-negative and HPV-positive HNSCC were included. In addition, a search in Clinicaltrials.gov for registered and ongoing trials in patients with HNSCC, including OPSCC, was performed. From these articles a selection was made to discuss the insights and developments in OPSCC immunity, biomarkers, and immunotherapy within the last decade. Last but not least, these studies were supplemented with general literature to explain concepts.

## The Tumor Immune Microenvironment

The TME plays a pivotal role in the clinical behavior and response to different sorts of therapy of various cancer types (30). In-depth studies on the type, balance and interaction of immune cells in the TME and how this may affect clinical behavior could open the door to rationally designed strategies to treat patients with OPSCC (23).

### Lymphocytes

The influx of high number of T cells was positively associated with clinical outcome of OPSCC after standard of care therapy (23, 31–33). In particular, strong infiltration by CD8+ and CD4+ T cells in pretreatment OPSCC tissues was associated with lower T stage, improved disease specific survival (DSS) and prolonged overall survival (OS). The clinical benefit of tumor infiltration with T cells was irrespective of HPV status of the tumor, albeit that HPV-induced tumors were more often strongly infiltrated by these T cells (31, 34–38). The functional activity of these tumor-infiltrating T cells is also important. Higher interferon gamma (IFNγ) and lower interleukin-4 (IL-4) and transforming growth factor-beta (TGF-β) cytokine expression levels were observed in HPV+ than HPV-negative OPSCC (39). In line with this, HPV-induced OPSCC contain high numbers of IFNγ or IL-17 producing CD8+ T cells (40) and the presence of IFNγ-producing CD4+ and CD8+ T cells in OPSCC, as inferred by the expression of the transcription factor T-box (Tbet) expressed in T cells, was related to better OS (6). Interestingly, the presence of IL-17-producing non-T cells in HPV+ OPSCC was associated with worse clinical benefit (38).

The analysis of freshly dissociated OPSCC tissues by mass cytometry revealed that HPV16+ OPSCC, comprising HPV16-specific T cells, also contained high numbers of CD161+ classical T cells, CD103+ tissue resident CD8+ T cells, dendritic cells (DCs) and DC-like macrophages (6). CD161+ T cells were highly activated, as shown by the high expression levels of PD-1, CD38 and HLA-DR, and were shown to be superior effector cells as they produced more IFNγ per cell (24). Notably, a higher frequency of CD161+CD4+ T cells was found to be associated with prolonged survival in OPSCC (24). The abundant presence of CD103+CD8+ T cells in HPV16+ OPSCC and its correlation with better prognosis was also demonstrated by others (41, 42). These CD103+CD8+ T cells represent non-circulating memory T cells that play a key role in local immunosurveillance (43) and are enriched for a number of genes associated with tissue resident cells (44). Single cell RNA analysis of 13 OPSCC revealed that these cells also expressed genes associated with cytotoxic potential, exhaustion/co-inhibition programmed cell death protein 1 (PD-1), lymphocyte activation gene 3 (LAG3), and T cell immunoglobulin and mucin domain-containing protein 3 (TIM3)) as well as activation/co-stimulation [CD27, inducible T cell costimulator (ICOS) and tumor necrosis factor receptor superfamily member 14 (TNFRSF14)] (45).

An important aspect in tumor control by T cells is the interaction with human leukocyte antigen (HLA) molecules presenting tumor-derived antigens at the cell surface of tumor cells, yet HLA expression is often lost or decreased in OPSCC (46, 47). Antigen presentation to CD8+ T cells may also be impaired by alterations in antigen processing pathway components such as the endoplasmic reticulum aminopeptidase 1 (ERAP1), an enzyme involved in trimming the N-terminus of peptide until it fits in the HLA class I molecule. Some polymorphisms in ERAP-1 were associated with high or low T cell infiltration of OPSCC. Interestingly, only the ERAP1 allotypes present in highly infiltrated HPV16+ OPSCC were capable of trimming model antigens, including HPV16 E7, to the proper HLA class I binding epitope (48). In contrast to HPV-negative OPSCC, there is no correlation between HLA class I expression and survival for HPV+ OPSCC (49, 50). The expression of HLA class II, which was more often found on tumor cells in HPV+ OPSCC, is associated with longer OS, DFS and disease specific survival (DSS) in OPSCC (46), supporting a role for CD4+ T cells in the control of OPSCC.

### The Curious Relationship Between Regulatory T Cells and OPSCC Survival

Regulatory T cells (Tregs) act as gatekeepers of immunological tolerance, and dysfunction in Treg-mediated control plays an important role in autoimmune and allergic disorders and cancer. Tregs play a dismal role in tumor immunity, with numerous studies revealing their role in suppressing anti-tumor immune responses and promoting tumor progression in various types of cancer. In HNSCC, including OPSCC, there is conflicting evidence regarding their role in suppressing tumor immunity and survival (51). In two small scale studies, a negative impact on clinical outcome and disease progression was observed for high frequencies of Tregs in the peripheral blood of patients with OPSCC (52, 53), whereas other studies including OPSCC demonstrated no impact of tumor-infiltrating Tregs (54) or a positive impact of circulating or tumor-infiltrating Tregs (38, 55, 56) on clinical outcome and/or survival. A similar debate has been described for colorectal cancers (57, 58), resulting in the definition of three populations of CD4+Foxp3+ T cells, namely highly suppressive CD45RA-negative Foxp3^hi^ effector Tregs (Foxp3^hi^ eTregs), suppressive CD45RA+Foxp3^int^ naïve Tregs (Foxp3^int^ nTregs), and non-suppressive cytokine-producing CD45RA-negative Foxp3^int^ T cells (59–61). Patients with colorectal tumors that were predominantly infiltrated with non-suppressive and cytokine-producing CD45RA-negative Foxp3^int^ T cells displayed a much better survival than patients with tumors mainly infiltrated with suppressive Foxp3^hi^ eTregs (60). This phenomenon may also play a role in studies that evaluated the clinical significance of intratumoral Tregs in OPSCC by means of immunohistochemistry (IHC), since discrimination between Foxp3^int^ and Foxp3^hi^ is difficult using this technique.

Part of the controversy on the prognostic value of Tregs may also be attributed to differences in the type of samples assessed, i.e. peripheral blood versus tumor tissues, and the absence of knowledge about the HPV status of the assessed tumors (51). The latter may be important as HPV-associated OPSCC is a distinct clinical entity with a different intratumoral immune cell make-up and a much better prognosis after (chemo)radiotherapy than HPV-negative OPSCC, particularly in patients with a concomitant HPV-specific and type 1-oriented intratumoral T cell response (6, 14, 32, 62).

Alternatively, differences in the effects of Tregs on clinical outcome may also come from their functional adaptability. Tregs can mirror effector cells by adopting the transcriptional profile of the cells they aim to suppress. In mice, Foxp3+ Tregs have been described to upregulate signal transducer and activator of transcription 3 (STAT3), GATA binding protein 3 (GATA-3)/interferon regulatory factor 4 (IRF4), or Tbet to control T helper type 17 (Th17), Th2 or Th1 inflammatory responses, respectively, in persistent infection and autoimmunity (63–66). We recently described that Tbet+ Tregs accumulate in tumors of HPV16+ OPSCC patients, and that their presence was associated with prolonged survival following standard of care therapy (67). Albeit detected at lower levels, the number of infiltrating Tbet+ Tregs strongly correlated with the number of tumor-infiltrating Tbet-positive CD4+ and CD8+ effector T cells as well as with the detection of HPV16-specific T cells, both of which we previously demonstrated to be associated with better tumor control on standard of care therapy. These data suggested that Tbet expression within the intratumoral Treg population and the association with prolonged survival is merely the reflection of an ongoing beneficial Th1-oriented T cell response, and that the balance between pro- and anti-tumor T cells eventually determines clinical outcome.

### Myeloid Cells

The myeloid cell compartment constitutes another major player in the TME. One of the most abundant types of myeloid cells in the TME are macrophages. High numbers of tumor associated macrophages (TAMs) in the TME is most often associated with poor prognosis (68, 69). Macrophages are highly plastic and display a functional status ranging between classically activated anti-tumor type 1 macrophages (M1) and the alternatively activated tumor promoting type 2 macrophages (M2) (70). Incoming monocytes from the blood into the tumor can differentiate into macrophages dependent on the different cytokines they sense and the interactions they have with other cells present in the TME. While an inflamed TME with IFNγ results in M1 differentiation, high levels of interleukin 4 (IL-4) and IL-13 turn them into M2 (71). M2 can produce cytokines, such as TGF-β, vascular endothelial growth factor (VEGF), IL-6, IL-10, and prostaglandin E2 (PGE2), and chemokines that promote angiogenesis or destruct the extracellular matrix, thereby helping tumor cells to invade and metastasize and they can stimulate Treg development and expansion while suppressing CD8+ T cell function (72, 73). Moreover, in response to inflammation (IFNγ production) or hypoxia TAMs can upregulate inhibitory receptor ligands for T cells like PDL-1 and PDL-2 (69, 72).

CD68+CD163+ M2 macrophages are often detected in OPSCC (46, 74). A higher infiltration with these macrophages was associated with poor clinical outcome in two studies on HNSCC tumors with the majority or all being OPSCC (46, 75). The strong stromal presence of CD68+CD163+ macrophages was associated with shorter DFS, DSS and OS, independent of HPV status in OPSCC (46), but this does not mean that all macrophages are bad as high infiltration with macrophages, the minority being M2, was associated with better PFS in p16+ OPSCC (47). This suggests that other macrophages may benefit tumor control. Indeed, in various tumor types high infiltration with M1 (CD14+CD163-negative) macrophages is associated with prolonged survival (76, 77). It also implies that simply depleting all macrophages in the TME may have detrimental effects and even accelerate tumor growth or increase recurrences (72, 78, 79). Therefore, reprogramming TAMs is currently under investigation in solid tumors, to move the balance from pro-tumorigenic towards tumor fighting macrophages (72, 80) and it is highly likely that also a strong Th1 cell response may aid in this process (73).

Similar to TAMs also myeloid derived suppressor cells (MDSCs) can be recruited from the blood or generated locally by arresting monocytes in their immature state. The phenotype of these MDSC is often described as CD14+ HLA-DR-CD33+CD11b+ (81, 82), hence monocytic MDSC (mMDSC). High circulating numbers of these cells are associated with metastasis and recurrences in OPSCC patients (83). Interestingly, treatment with tadalafil, a phospohodiesterase 5 inhibitor to block nitric oxide and arginase 1 production by myeloid cells, resulted in the activation of a stronger tumor-specific T-cell response with no difference in responsiveness between HPV16+ OPSCC and HPV-negative (non)oropharyngeal cancer patients (84). Several other studies to deplete mMDSCs, prevent their recruitment or block their suppressive function have been undertaken (85). Next to these mMDSC also granulocytic MDSC (gMDSC) and neutrophils may enter the tumor to suppress effector T cells in the TME. However, no studies on the role of these cells in the TME of OPSCC have been reported.

Finally, recruited monocytes can also differentiate into DCs. High numbers of CD14+CD11b+CD11c+ DC-like macrophages and CD14-negative CD11b^dim^CD11c+ DCs have been detected in the TME of OPSCC patients with a good clinical outcome (6). In another study, high numbers of stromal CD11c+ DC was an independent prognostic factor for better survival in HNSCC, including OPSCC (55). Current studies have analyzed DCs by only a few markers. Recently, two in-depth studies on blood derived DCs have divided these in several phenotypic and functional subtypes (86, 87). We applied high-dimensional flow cytometry and multispectral imaging to reveal a new subset of DCs, called CD163+ cDC2 or DC3, the presence of which was directly related to survival in HPV16+ OPSCC (74). Moreover, these DCs displayed the capacity to specifically trigger type 1 T cell responses by their production of IL-12 and IL-18 (74) and may be involved in conferring a tissue-resident signature to stimulated T cells (88).

### Expression of Immune Checkpoints

Intratumoral T cell reactivity is regulated *via* so-called co-stimulatory and co-inhibitory (or checkpoint) molecules. Well-known is the suppression of T cells expressing PD-1 *via* PD-1 ligand (PD-L1) and blockade of this axis has resulted in spectacular clinical responses for a number of tumor types. Analyses of checkpoint expression in OPSCC revealed that the expression of PD-1 and/or PD-L1 was related to a stronger immune infiltration and good prognosis after standard therapy (89–92), most likely as it reflects an ongoing immune response in which type I and II interferons are produced. The presence of intratumoral PD-L1 expressing CD68+ macrophages and CD8+ T cells was found to be associated with improved OS (93). In addition, rich immune infiltration, comprising PD1+CD8+ T cells and CD68+ macrophages, was found to be associated with a better clinical response to checkpoint therapy (94). While the numbers of infiltrating total CD8+ T cells and CD68+ macrophages were higher in HPV+ OPSCC, the percentage of CD8+PD-1+ T cells was similar, and the percentage of CD68+ PD-L1+ macrophages lower in HPV+ OPSCC compared to HPV-negative OPSCC (95).

Another actionable co-inhibitory molecule is natural killer group 2 member A (NKG2A) (96, 97), which together with its co-receptor CD94 is expressed by many of the tumor-infiltrating CD8+ T cells and only by a minority of the CD4+ T cells in OPSCC (45). Remarkably, NKG2A expression on CD8+ in OPSCC is independent from PD-1 and often found on CD103+ early effector tissue resident CD8+ T cells (45, 97). The frequency of intratumoral NKG2A/CD94+ CD8+ T cells was higher in in HPV16+ OPSCC patients with a demonstrable ongoing HPV16-specific T cell response when compared to HPV16+ OPSCC lacking such an anti-tumor response or to HPV-negative OPSCC patients (6, 97). NKG2A interacts with HLA-E, which is a non-classical highly-conserved HLA class I molecule that is expressed by many cancers (96, 98, 99), including OPSCC (50). The interaction between NKG2A and HLA-E is thought to block the cytotoxic activity of CD8+ T cells and NK cells (100) and a couple of studies have shown that expression of HLA-E by tumor cells restrained the prognostic impact of tumor-infiltrating CD8+ T cells (98, 99), including that of HPV16+ OPSCC (97).

Other inhibitory receptors found to be upregulated on activated T cells in the TME of OPSCC include TIM3, LAG3 and T cell immunoreceptor with Ig and ITIM domains (TIGIT) and others (45). All expressed on higher numbers of T cells in HPV+ when compared to virus-negative head and neck tumors, but only in HPV+ tumors each of these markers was associated with prolonged survival (101).

Overall, the expression of inhibitory receptors are more indicative for an inflamed TME with ongoing antitumor immunity than for an exhausted T cell response in OPSCC. Nevertheless, the interaction between inhibitory receptors and their ligands will inhibit the activation and effector functions of T cells impairing their capacity to control OPSCC growth.

## The Blood Compartment for Biomarker Analysis

An important question is whether the TME biomarkers associated with clinical outcome are also detectable and prognostic when analyzed on immune cells present in blood, as this compartment is easily accessible and allows for kinetic studies. The easiest approach is to determine differential leukocyte counts on blood samples, which is used in all hospitals as a normal diagnostic routine. High neutrophil counts in OPSCC, and more specifically high neutrophil-to-lymphocyte ratio (NLR) in the blood sample prior, during and after radiotherapy correlated with poor OS, recurrence free survival (RFS) and/or DSS as well as distant metastasis (102–105). Also, in HPV16+ OPSCC patients, a high NLR in the blood sample obtained prior to concurrent chemoradiation correlated with decreased OS. Neutrophils appear to have an unique phenotype of immature granulocytes CD11c^bright^/CD62L^dim^/CD11b^bright^/CD16^bright^, which are absent in blood of healthy donors (106).

High circulating monocyte counts prior to therapy also correlated to reduced OS and RFS in HPV16+ OPSCC patients (102, 107). While monocyte counts in HPV-negative OPSCC patients were even higher, the absolute monocyte counts were not related to clinical outcome in this patient group (102), suggesting that these monocytes might have a negative impact only in highly immunogenic tumors. Potentially, the composition of the monocyte subtypes may be of importance as some of them may constitute CD14+ MDSC, which have shown to exhibit immune suppressive effects (6, 108). Furthermore, the percentage of classical (CD14^high^CD16-negative) monocytes were shown to be increased in OPSCC patients as well as expressed PD-L1 (107). Also a high monocyte-to-lymphocyte ratio was associated with lower OS in OPSCC (107, 109).

So far, no comparative studies have been performed with respect to neutrophils and MDSC in order to analyze if the blood compartment reflects the TME.

The relation between higher numbers of tumor-infiltrating T cells and clinical outcome prompts the question if this can also be detected in the blood. High pre-treatment numbers of CD8+ T cells in the blood correlated with improved OS in HPV16+ OPSCC patients (14). However, absolute CD4 counts as well as frequencies of CD4+ T cells measured in blood of OPSCC was not correlating to clinical outcome (14). This seems logical as the population of CD4+ T cells comprises naïve T cells, type 1 and type 2 effector T cells, memory T cells and also Tregs which dilutes the real impact of one of these subpopulations. Moreover, the peripheral blood levels of CD4+ and CD8+ T cells were not related to the type and degree of specific T cell subset infiltration in OPSCC (34). Interestingly, it was found that HPV+ OPSCC with elevated Treg frequencies in the blood displayed a better OS than patients with HPV-negative tumors or patients with HPV+ OPSCC patients displaying relatively low numbers of circulating Tregs (56).

Last but not least, HPV16-specific T cells have been detected in the circulation of patients with OPSCC using functional assays in which PBMC were stimulated with the HPV-encoded antigens (110–112). The detection of such an HPV16-specific T cell response in the peripheral blood did not always coincide with the presence of these HPV16-specific T cells in the TME, this (110). HPV16-specific T cells could also be detected in a large percentage of healthy individuals (113–115) indicating that their presence is not enough to function as a biomarker.

All together there are a couple of opportunities (neutrophils, monocytes, Tregs) that may be exploited as blood biomarkers reflecting the TME, but more sophisticated measurements of subpopulations and validation of the used techniques are warranted.

## The Role of Tumor-Specific T Cells in the TME of OPSCC

There is ample evidence that tumors highly infiltrated with CD4+ and/or CD8+ T cells (also referred to as immunologically “hot” tumors) respond better to (immuno-)therapy (27, 116). In OPSCC, HPV infection may play a very important role in this phenomenon. Superior prognosis of HPV+ OPSCC over HPV-negative OPSCC following chemoradiotherapy has been reported (13, 117). Interestingly, for the majority of HPV+ OPSCC this was related with a more dense and activated T cell infiltrate, suggesting a role for HPV-specific immunity in tumor control (14, 62, 118).

HPV16+ tumors express the virally derived oncoproteins E6 and E7, and it has been suggested that these “non-self” HPV antigens can evoke immune responses. Indeed, cellular and humoral immunity against these antigens can be detected in the peripheral blood and TME of HPV16+ OPSCC patients (110, 119–123). Notably, there is a direct link between the presence of an intratumoral HPV-specific T-cell response and the good prognosis of HPV16-driven OPSCC (6). Patients with an HPV16-driven tumor and a concomitant intratumoral HPV16-specific T cell response have a much better survival after (chemo)radiotherapy than patients with an HPV16-driven tumor without such an HPV16-specific T cell response or patients with HPV-negative OPSCC. Moreover, the presence of these HPV-specific T cells was associated with a type 1 oriented TME and a much higher level of activated CD161-expressing effector memory CD4+ T cells,CD103+ tissue-resident CD8+ T cells, and tissue-resident memory T cell-stimulating CD163+ cDC2 (6, 74).

Although a clear relation could be found between the dense, type 1 oriented HPV-specific immune infiltrate and disease outcome in HPV16-driven OPSCC, there is also evidence for improved survival of HPV-negative OPSCC patients with strongly T-cell infiltrated tumors (37, 124). Unfortunately, knowledge on the tumor-specificity is lacking in these studies. A promising and emerging field in studies of the anti-tumor response is the recognition of tumor neoantigens. Tumors with high numbers of nonsynonymous mutations have a greater likelihood of presenting mutation-derived neoepitopes and consequently mounting a T-cell response against these epitopes thereby improving the clinical response (125). Tumor mutational burden (TMB) in HNSCC is relatively high and comparable to other smoking related tumor types. Viral HNSCC display only half of the mutations rate observed in non-viral HNSCC (125, 126). CD8+ T cells responding to such mutations have been detected in a few patients with non-OPSCC either spontaneously induced (127) or following a complete response after pembrolizumab treatment (128). Notably, such neoantigen specific T cells can also be detected in low TMB tumors (129), suggesting that they may also be present in OPSCC.

## Immunotherapy in OPSCC

During the last two decades, several strategies aiming to boost the immune response to cancer have been developed and tested in patients with cancer, ranging from immune modulators to checkpoint therapy, adoptive cell transfer, and vaccines. **Table 1** summarizes active/recruiting immunotherapy trials in patients with OPSCC and HNSCC, including OPSCC.

**Table 1:** Immunotherapy in patients with OPSCC.

NCT number	Phase	Number of arms	Randomized	Treatment	Target	Setting	Previous therapy	Number of patients	Endpoint	Trial Status
OPSCC	Immune modulators									
2002182	2	2	No (open label)	ADXS11-0001 vs control (SoC)	ADXS11-0001: HPV16 E7	Neoadjuvant (pre-surgery)	None	30 HPV+	Safety + IR	Active (Not recruiting)
4106362	2	2	Yes (open label)	RT-CT + Cetuxi vs RT-CT	Cetuxi: anti EGFR	First line	None	70 HPV+ KRA variant+ stage III–IV	Efficacy + toxicity	Recruiting
4508829	2	1	No (open label)	IMRT + CT + anti EGFR moAb	Anti EGFR	Concurrent	SoC (no RT)	52 (advanced stage)	Efficacy	Recruiting
OPSCC	Checkpoints (+ combinations)									
3144778	1	2	Yes (open label)	Durva vs Durva + Treme	Durva: anti PD-L1 Treme: anti CTLA-4	Durva + Treme prior to surgery	None	28 stage II–IVA	Safety + IR	Active (not recruiting)
3618134	1/2	1	No (open label)	RT + Durva vs RT + Durva + Treme	Durva: anti PD-L1 Treme: anti CTLA-4	Prior to surgery	None	82 HPV+ stage I–III	Safety + efficacy	Recruiting
3715946	2	1	No (open label)	RT (reduced) + Nivo	Nivo: anti PD-1	Adjuvant	Surgery	135 (primary tumor)	Efficacy	Recruiting
3799445	2	1	No (open label)	Ipi + Nivo + RT	Ipi: anti CTLA-4	First line	None	180 HPV+ stage I–IVA	Safety + efficacy	Recruiting
				Nivo: anti PD-1						
3838263	2	2	Yes (2:1; open label)	Nivo vs CRT (control)	Nivo: anti PD-1	Nivo before RT-CT	None	61 HPV+	Safety + efficacy	Recruiting
3811015	2/3	3 (incl. cross over)	Yes (open label)	IMRT + CT + Nivo vs IMRT + CT (control)	Nivo: anti PD-1	Nivo post RT-CT	None	744 HPV+	Efficacy + Prognostic biomarker	Recruiting
3952585	2/3	3	Yes (open label)	IMRT + CT vs IMRT (reduced) + CT vs Nivo + IMRT (reduced) + CT	Nivo: anti PD-1	Nivo prior to IMRT(reduced) + CT	None	711 p16+	Efficacy	Recruiting
OPSCC	ACT									
4015336	2	1	No (open label)	E7 TCR T cells	E7 TCR T cells: HPV16 E7	HLA-A0201	None	180 HPV16+ stage II–III	Safety	Recruiting
OPSCC	Vaccines (+ combinations)									
3258008	2	1	No (open label)	Utomi + ISA101b	Utomi: agonistic CD137	Adjuvant	SoC	27 HPV+	Efficacy + toxicity	Active (not recruiting)
					ISA101b: HPV16 E6E7					
3669718	2	2	Yes (Double blinded)	ISA101b + Cemip vs Cemip	ISA101b: HPV16 E6E7	Adjuvant	SoC	194 HPV16+ R/M	Efficacy + toxicity	Recruiting
					Cemip: anti PD-1					
4001413	2	2	Yes (open label)	Durva vs Durva + MEDI0457	Durva: anti PD-L1	Adjuvant	SoC	66 HPV+	Efficacy + toxicity	Recruiting
					MEDI0457: HPV16/18 E6E7					
HNSCC	Immune modulators									
3088059	2	Multiple	No (open label)	Afatinib, Palbociclib, Niraparib, Rogaratinib (BAY1163877)	Afatinib: kinase inhibitor	Adjuvant	CT	340 R/M	Efficacy + Biomarker	Recruiting
					Palbociclib: kinase inhibitor					
					Niraparib: PARP inhibitor					
					Rogaratinib: FGFR tyrosine kinase inhibitor					
3795610	2	1	No (open label)	IPI-549	IPI-549: PI3Ky inhibitor	Neoadjuvant (pre-surgery)	None	15 advanced	PI3Ky changes + IR + toxicity	Recruiting
HNSCC	Checkpoints (+ combinations): Ipilimumab									
1935921	1	1	No (open label)	Ipi + cetuxi +RT	Ipi: anti CTLA-4	Concurrent	None	19 stage III–IV	Dose finding + efficacy + biomarkers	Active (not recruiting)
					Cetuxi: anti EGFR					
HNSCC	Checkpoints (+ combinations): Nivolumab									
2741570	3	2	Yes (open label)	Nivo + ipi vs SoC	Nivo: anti PD-1	First line	None	947 R/M	Efficacy	Active (not recruiting)
					Ipi: anti CTLA-4					
2764593	1	4	No (open label)	Nivo + CT + IMRT, Nivo + high CT + IMRT, Nivo + Cetuxi + RT, Nivo + IMRT	Nivo: anti PD-1	Neoadjuvant (pre-RT)	None	40 stage I–IV	Toxicity	Active (not recruiting)
					Cetuxi: anti EGFR					
2823574	2	2	Yes (double blinded)	Nivo + ipi vs nivo (control)	Nivo: anti PD-1	Adjuvant	None	675 R/M	Efficacy	Active (not recruiting)
					Ipi: anti CTLA-4					
2834013	2		No (open label)	Nivo + ipi vs nivo (control)	Nivo: anti PD-1	Adjuvant	SoC	818 PD (rare tumors)	Efficacy + toxicity	Recruiting
					Ipi: anti CTLA-4					
3247712	1/2	4	No (open label)	Nivo + RT	Nivo: anti PD-1	Neoadjuvant (pre-surgery)	None	28 (eligible for surgery)	Safety + feasibility + efficacy	Recruiting
3317327	1/2	1	No (open label)	Nivo	Nivo: anti PD-1	Neoadjuvant (pre-RT)	SoC	20 R	Safety + efficacy	Recruiting
3341936	2	1	No (open label)	Nivo + Liri	Nivo: anti PD-1	Neoadjuvant (pre-salvage surgery)	SoC	58 R	Safety + efficacy	Recruiting
					Liri: anti KIR2DL1/2L3					
3406247	2	2	No (open label)	Nivo alone vs nivo + ipi	Nivo: anti PD-1	Adjuvant	Salvage surgery after RT	140 P/R	Safety + efficacy	Not yet recruiting
					Ipi: anti CTLA-4					
3620123	2	2	Yes (open label)	Nivo + ipi vs docetaxel	Nivo: anti PD-1	Palliative	SoC	280 R/M	Efficacy	Recruiting
					Ipi: anti CTLA-4					
3854032	2	2	Yes (open label)	Nivo + BMS-986205 vs Nivo	Nivo: anti PD-1	Neoadjuvant (pre-surgery)	None	48 stage II–IV (non R)	Efficacy + IR + toxicity	Recruiting
					BMS-986205: IDO1 inhibitor					
4080804	2	3	Yes (open label)	Nivo + ipi vs nivo + Relatlimab vs nivo	Nivo: anti PD-1	Neoadjuvant (pre-surgery)	None	60 advanced	Safety + IR + efficacy	Recruiting
					Ipi: anti CTLA4					
					Relatlimab: anti LAG3					
HNSCC	Checkpoints (+ combinations): Pembrolizumab									
2296684	2	1	No (open label)	Pembro	Pembro: anti PD-1	Neoadjuvant (pre-surgery)	None	66 stage III–IV	Efficacy + toxicity	Recruiting
2586207	1	1	No (open label)	Pembro + CRT	Pembro: anti PD-1	Concomitant	SoC	57 stage III–IV	Safety + QoL	Active (not recruiting)
2707588	2	2	Yes (open label)	Pembro + RT vs Cetuxi + RT	Pembro: anti PD-1	Concomitant	SoC	133 advanced	Efficacy + toxicity + QoL +impact	Active (not recruiting)
				Cetuxi: anti EGFR	p16/HPV					
2718820	1/2	1	No (open label)	Pembro + docetaxel	Pembro: anti PD-1	Adjuvant	SoC	22 R/M	Efficacy + QoL + toxicity	Active (not recruiting)
2769520	2	1	No (open label)	Pembro	Pembro: anti PD-1	Adjuvant	Surgery	45 R	Efficacy + toxicity	Recruiting
2775812	1	1	No (open label)	Pembro + CT + IMRT	Pembro: anti PD-1	Concomitant	SoC	37 stage III–IV (high risk)	RP2D + efficacy + toxicity + biomarkers	Active (not recruiting)
					CT: cisplatin					
2777385	2	2	Yes (open label)	Pembro + CT + IMRT	Pembro: anti PD-1	Adjuvant	SoC	90 (non M)	Efficacy + toxicity	Recruiting
					CT: cisplatin					
2819752	1	2	No (open label)	Pembro + CRT	Pembro: anti PD-1	Concomitant	SoC	36 stage IV (HPV+ vs HPV-)	MTD + toxicity + efficacy	Active (not recruiting)
2841748	2	2	Yes (double-blinded)	Pembro vs placebo	Pembro: anti PD-1	Adjuvant	SoC	100 stage III–IV (high risk)	Efficacy	Recruiting
3082534	2	4	No (open label)	Pembro + Cetuxi	Pembro: anti PD-1	Concurrent	SoC	83 R/M	Efficacy	Recruiting
					Cetuxi: anti EGFR					
3383094	2	2	Yes (open label)	Pembro + IMRT vs IMRT + CT (control)	Pembro: anti PD-1	Concurrent + adjuvant	None	114 p16+ stage III–IV	Efficacy + toxicity	Recruiting
3546582	2	2	Yes (open label)	Pembro + RT vs RT	Pembro: anti PD-1	Adjuvant	RT	102 R or second primary	Safety + efficacy	Recruiting
3695510	2	1	No (open label)	Afatinib + Pembro	Afatinib: kinase inhibitor	Adjuvant	SoC	29 R/M	Efficacy + toxicity	Recruiting
					Pembro: anti PD-1					
HNSCC	Checkpoints (+ combinations): Pembro									
4193293	1/2	1	No (open label)	Duvelisib + pembro	Duvelisib: PI3K inhibitor	Adjuvant	SoC	30 R/M	DLT + efficacy + safety	Recruiting
					Pembro: anti PD-1					
HNSCC	Checkpoints (+ combinations): Durvalumab									
2551159	3	3	Yes (open label)	Durva vs durva + treme vs SoC	Durva: anti PD-L1	First line	None	823 R/M	Efficacy + PK + IR + QoL	Active (not recruiting)
					Treme: anti CTLA-4					
2827838	2	1	No (open label)	Durva	Durva: anti PD-L1	Neoadjuvant (pre-surgery)	None	20 stage I–IV	IR vs HPV status	Recruiting
2997332	1	1	No (open label)	Durva + CT	Durva: anti PD-L1	Adjuvant	SoC	36 advanced	Safety + RP2D	Recruiting
3051906	1/2	1	No (open label)	Durva + cetuxi + RT	Durva: anti PD-L1	Adjuvant	SoC	69 advanced	Efficacy + toxicity	Not yet recruiting
					Cetuxi: anti EGFR					
3088059	2	Multiple	No (open label)	SoC, IPH2201 or durva	IPH2201: anti NKG2A	Adjuvant	CT	340 R/M	Efficacy + Biomarker	Recruiting
					Durva: anti PD-L1					
3258554	2/3	2	Yes (open label)	RT + durva vs RT + cetuxi	Durva: anti PD-L1	Adjuvant	SoC	523 stage III–IV	DLT + efficacy +QoL	Recruiting
					Cetuxi: anti EGFR					
3426657	2	1	No (open label)	Durva + treme + + CT + RT	Durva: anti PD-L1	Adjuvant	CT	120 advanced	Feasibility + IR + toxicity + efficacy	Recruiting
					Treme: anti CTLA-4					
3509012	1	8	No (open label)	Durva + treme + CRT	Durva: anti PD-L1	Adjuvant	SoC	360 advanced	Toxicity + efficacy	Active (not recruiting)
					Treme: anti CTLA-4					
3529422	2	1	No (open label)	Durva + Treme + RT	Durva: anti PD-L1	Adjuvant	Surgery	24 stage III–IV (non M)	Safety + efficacy	Recruiting
					Treme: anti CTLA-4					
HNSCC	Checkpoints (+ combinations): others									
2999087	3	4	Yes (open label)	Avelu + cetuxi + RT vs cetuxi + RT vs CRT	Avelu: anti PD-L1		None	688 advanced	Efficacy	Recruiting
					Cetuxi: anti EGFR					
4129320	2/3	4	Yes (open label)	Enoblitu + MGA012 vs Enoblitu + MGA012 + CT vs MGA012 + CT vs Pembro + CT (control)	Enoblituzumab: anti B7H3 (MGA271)		None	750 R/M	Efficacy + toxicity + IR + QoL	Not yet recruiting
					MGA012: anti PD-1					
					Pembro: anti PD-1					
2274155	1	3	No (open label)	MEDI6469	MEDI6469: OX40 agonist	Neoadjuvant (pre-surgery)	None	17 advanced	Safety + feasibility + IR + efficacy	Active (not recruiting)
HNSCC	ACT									
3083873	2	5	No (open label)	LN-145 non-cryopreserved vs cryopreserved	autologous TIL	Adjuvant	SoC	55 R/M	Efficacy + safety	Recruiting
3578406	1	2	Yes (open label)	TCR-T with or without anti PD-1 secreting element	HPV16 E6-specific T cells	Adjuvant	SoC	20 HPV16+ R/M	MTD	Recruiting
HNSCC	Vaccine (+ combinations)									
1998542	2	1	No (open label)	AlloVax + CRCL + AlloStim	AlloVax	Adjuvant	SoC	12 R/M	Safety + efficacy	Completed
					CRCL: chaperone rich cell lysate					
					AlloStim: adjuvant					
2865135	1b/2	1	No (open label)	DPX-E7	DPX-E7:HPV16 E7_1-19_	Adjuvant	SoC	44 HPV+ HLA-A*02+	Safety + efficacy	Active (not recruiting)
3162224	1b/2a	1	No (open label)	MEDI0457 + Durva	MEDI0457: HPV16/18 E6E7	Adjuvant	SoC	35 HPV+ R/M	Safety + efficacy	Active (not recruiting)
					Durva: anti PD-L1					
3260023	1b/2	1	No (open label)	TG4001 + Avelu	TG4001: HPV16 E6E7	Adjuvant	SoC	52 HPV+ R/M	Safety + DLT (+ efficacy in phase II)	Recruiting
					Avelu: anti PD-L1					
3633110	1/2	2	No (open label)	GEN-009 vs GEN-009 + anti PD-1	GEN-009: neoepitope SLP vaccine	Adjuvant	SoC	99 NED	Safety + IR + efficacy	Active (not recruiting)
					Anti PD-1: nivo or pembro					
3946358	2	1	No (open label)	UCPVax + Atezo	UCP: telomerase derived	Adjuvant	SoC	47 HPV+ advanced/M	Efficacy + QoL	Recruiting
4180215	1/2	Multiple	No (open label)	HB-201 (IV or IT) vs HB-201 + CI	HB-201: HPV16 E7E6	Adjuvant	SoC	100 HPV+	Dose finding + toxicity	Recruiting
4287868	1/2	2	No (open label)	PDS0101 + M7824 + NHS-IL12	PDS0101: HPV	Adjuvant	SoC	40 HPV+ advanced/M	Safety + efficacy	Recruiting
					M7824: anti PD-L1/TGFβ					
					NHS-IL12: IL12					
4432597	1/2	4	No (open label)	PRGN-2009 vs PRGN-2009 + M7824	PRGN-2009: HPV	Neoadjuvant or induction	SoC	70 HPV+ R/M	Safety + efficacy	Recruiting
					M7824: anti PD-L1/TGFβ					

Clinical trials studying immunotherapy in patients with OPSCC and HNSCC (including OPSCC). Afatinib, protein kinase inhibitor, highly selective irreversible ErbB family blocker (including EGFR); AlloStim, living cell product, consisting of activated allogeneic type 1 CD4^+^ memory T cells containing cytolytic activity and have the following phenotype CD45RO^hi^, CD62L^lo^, CD40L^hi^, CD25^+^, IFN-gamma^+^, IL-4^-^, granzyme^+^, and perforin^+^, and is used as adjuvant; Avelu, avelumab (anti PD-L1 antibody); Cemip, Cemiplimab REGN2810 (anti PD-1 antibody); Cetuxi, cetuximab (anti EGFR antibody); CRCL, chaperone rich cell lysate as source of tumor antigens prepared from autologous tumor; CRT, chemoradiation therapy; CT, chemotherapy; CTLA-4, cytotoxic T lymphocyte antigen 4; DLT, dose limiting toxicity; Durva, Durvalumab (anti PD-L1 antibody); EGFR, epidermal growth factor receptor; Enoblitu, Enoblituzumab (anti B7H3/CD276 antibody); FGFR, fibroblast growth factor receptor; HB-201, arenavirus vector-based vaccine expressing inactivated fusion protein HPV16 E7E6; HLA, human leukocyte antigen; HNSCC, head and neck squamous cell carcinoma (including OPSCC); HPV, human papillomavirus; IDO-1, indoleamine 2,3-dioxygenase-1; IMRT, intensity modulated radiotherapy; IPH2201, Monalizumab (anti NKG2A antibody); Ipi, ipilimumab (anti CTLA-4); IR, immune response; KIR, killer cell immunoglobulin-like receptor; LAG-3, lymphocyte activation gene 3; Liri, Lirilumab (anti KIR2DL1/2L3 antibody); LN-145, Lifileucel, autologous TIL adoptively transferred with addition of IL-2 in patients receiving a nonmyeloablative lymphocyte depletion; M, metastatic cancer patients; M7824, bintrafusp alfa, fusion protein, bispecific antibody directed to TGF-beta and PD-L1; MEDI0457, INO-3112 HPV DNA vaccine; MoAb, monoclonal antibody; MTD, maximum tolerated dose; NED, no evidence of disease anymore after treatment; Nivo, Nivolumab (anti PD-1 antibody); NKG2A, natural killer group 2A inhibitory receptor, ligand of HLA-E; OPSCC, oropharyngeal squamous cell carcinoma; P, primary tumor; PARP, poly ADP ribose polymerase enzyme; PD, progressive disease; PD-1, programmed cell death protein 1; Pembro, Pembrolizumab (anti PD-1 antibody); PD-L1, programmed cell death ligand 1; PI3K, Phosphatidylinositol-3-kinase; PK, pharmacokinetics; R, recurrent cancer patients; RT, radiotherapy; QoL, quality of life; RP2D, recommended phase 2 dose; R/M, recurrent/metastasis patients; RT-CT, radiotherapy-chemotherapy (standard therapy); SLP, synthetic long peptide; SoC, standard of care therapy; TCR, T cell receptor; TCR-T, engineered T cells bearing HPV E6 specific TCR and armed with PD-1 antagonist that will be secreted; TG-4001, Vaccinia vector (MVA)-HPV16E6/E7-IL2 vaccine; TIL, tumor infiltrating lymphocytes; Treme, Tremelimumab (anti CTLA-4); UCPVax, Universal cancer peptides (UCP2 and UCP4) derived from telomerase, a CD4 T helper type 1 inducer cancer vaccine; Utomi, utomilumab (agonistic anti CD137 antibody).

### Immune Modulators

One of the earliest immune stimulators tested is recombinant IL-2 (rIL-2), used to directly stimulate the activity of tumor-specific T cells. In a phase III trial with 200 patients rIL-2 was injected close to the ipsilateral lymph node, each day for 10 days before surgery and radiotherapy and then each month at the contralateral LN site for 1 year. The treatment had no significant side effects but improved the DFS (from 51 to 64%) and OS (from 55 to 73%) at 5 years (130). At a later stage, IRX-2 was developed to stimulate T cell activity. IRX-2 is a mix of purified cytokines obtained after stimulating peripheral blood mononuclear cells (PBMC) with phytohemagglutinin (PHA). In a phase IIa trial treatment naïve patients with HNSCC received subcutaneous IRX-2 injections near the TDLN for 10 days prior to surgical resection, which with the knowledge of today is an interesting choice as our studies showed that the great majority of TDLN comprise tumor-specific T cells even when they are hardly detectable in the tumor itself (24, 131). Injection of IRX-2 was associated with small radiological reductions in tumor size, including patients with OPSCC (132). In a first analysis, pre-and-post IRX-2 samples were analyzed and showed marked increases in CD3+ T cells and CD68+ macrophages (133). In a subgroup of 7 patients pre-and post-samples could be compared, showing potential increases in CD4+ T cells (6/7) and CD68 macrophages (4/7) as well as decreases in CD8+ T cells (5/7) patients after IRX-2 treatment. Unfortunately, HPV status was not determined but one could envision that most of the responders would have a HPV-positive tumor. Nanostring analyses using the PanCancer IO360 immune profiling panel confirmed the increases in CD4+ T cells and most markedly in DC as well as in the immune modulatory cytokines IL-6 and IL-10 (134). Another compound that indirectly could stimulate the tumor-specific T cell response is the toll-like receptor 8 (TLR8) agonist motolomid, which is known to activate monocytes, DCs and NK cells (135). Treatment of recurrent or metastatic HNSCC patients after the first 6 cycles of chemotherapy with weekly infusions of cetuximab with or without motolomid did not result in improved OS in a trial with n=195 patients. However, in a prespecified subgroup of n=83 OPSCC patients the motolomid arm displayed longer PFS and OS when the tumor was induced by HPV (136), suggesting that motolomid may have boosted the existing but probably weak HPV-specific T cell response in these patients.

Other compounds target immune suppressive cells that are active in the TME. For instance, metformin, which is widely used as a drug to manage diabetes type 2, has been described to have anti-cancer effects (137). In mice, metformin increased the number of tumor infiltrating CD8+ T cells, and the combination of metformin and a tumor vaccine improved the multifunctionality of the vaccine-induced CD8+ tumor infiltrating lymphocytes (TIL) (138). Another potential mechanism is the effect of metformin on Tregs, as it also inhibits mammalian target of rapamycin (mTOR). Metformin treatment for a minimum of 9 days before surgery of HNSCC, more than half being OPSCC, resulted in a strong decrease of intratumoral Foxp3+ Tregs and increase of stromal CD8+ T cells between pre-and post-treatment samples (n=16 HPV+ and n=20 HPV-negative), independent of HPV status (139). Potentially, metformin treatment may be interesting for HPV+ OPSCC patients with ongoing tumor immunity. As high numbers of Tregs have infiltrated those tumors (67), the use of metformin may tip the balance in favor of spontaneous anti-tumor responses in these patients, resulting in tumor shrinkage. Similarly, it may provide benefit in combination with other types of immunotherapy. Several new immune modulators are currently tested in the clinic (**Table 1**).

### Checkpoint Blockade Therapy

The use of antibodies that block the interaction between PD-1 and PD-L1 are successfully used in several types of immunogenic cancers with a high mutation rate (e.g. melanoma, lung cancer) or induced by the Merkel cell virus (140–142). Several studies have been reported on the efficacy of such antibodies for the treatment of HNSCC, including patients with OPSCC (143–147).

In CheckMate 141, a phase III trial, platinum-resistant patients with recurrent or metastatic tumors were treated with the anti-PD-1 antibody nivolumab. The response rate was 13.3% in the nivolumab group versus 5.8% in patients treated with standard of care. The objective response rate (ORR) to nivolumab was 15.9% in HPV+ patients while it was 8.0% in HPV-negative patients (143). The 2-year OS in this study was 16.9% in patients with PD-L1 expression, regardless of the HPV status (148). Nivolumab also induced clinical benefit in patients lacking PD-L1 expression, albeit in a lower proportion of patients (148). Interestingly, continued treatment of patients with nivolumab with slow progressing disease still resulted in clinical benefit, irrespective of the HPV status of the tumor. This was associated with a decrease in the percentage of PD-1+ Tregs in PBMC (149), suggesting that an ongoing immune response was present in these patients but which was simply too weak to be effective. Importantly, nivolumab treatment improved OS irrespective of prior treatment with cetuximab, an antibody known for its immune modulating side effects (150–153), albeit that the reduction in risk of death was lower in pre-treated patients (154). A similar response rate with PD-1 blockade using pembrolizumab was found in the KEYNOTE-012 phase Ib study of 60 patients with recurrent or metastatic tumors with PD-L1 expression. In total 18% of the patients displayed a clinical response, divided as 25% (4/16) in the HPV+ HNSCC patient group and 14% (4/29) in HPV-negative group (144).

The PD-L1 antibody durvalumab was tested in the expansion phase of a phase I/II study for treatment of patients with recurrent or metastatic tumors, 40% of which were HPV+. The ORR was 6.5% (4/62) in the total group with 15% in the PD-L1 expressing subgroup and 2.6% in the group with low PD-L1 expression. None of the HPV+ patients showed a response (145).

Another important checkpoint molecule is NKG2A. Preclinical experiments revealed that anti-NKG2A therapy promoted tumor immunity and synergized with PD-1 blockade. In addition, it was shown that NKG2A blockade improved the efficacy of tumor vaccines and adoptive cell therapy (ACT) (96, 97). The humanized NKG2A antibody monalizumab was tested together with cetuximab in previously treated recurrent or metastatic tumors, including 32% OPSCC. A preliminary report on the outcome of the trial revealed a confirmed clinical partial response (PR) in 8 out of 26 patients (31%) and stable disease (SD) in 14 out of 26 patients, which was regarded as promising when compared to historical cetuximab data (96).

In comparison to the high ORR in virus-driven Merkel cell carcinoma after checkpoint therapy (56% (142)), the response in HPV+ patients to checkpoint therapy is disappointingly low. However, in view of the fact that patients with both an HPV+ tumor and a strong immune response to HPV display an excellent response to standard of care therapy (6, 24), this result was to be expected. The majority of HPV+ patients with recurrent or metastatic tumors most likely are those in whom there was no or a very weak intratumoral T-cell response to HPV during first line treatment and it is known that checkpoint blockade works best in patients with a pre-existing tumor-specific immune response (155). Hence, in OPSCC these therapies should be combined with modalities that enhance the tumor-reactive T cell response in patients.

### Adoptive Cell Therapy

The infusion of large numbers of tumor-reactive T cells, being either ex-vivo expanded TILs, T-cell receptor (TCR) transduced T cells or chimeric antigen receptor (CAR)-T cells is one way to enhance the number of tumor-reactive T cells and has proven to be clinically effective in patients with advanced cancer (156–158). The presence of two strong tumor-specific antigens E6 and E7 in HPV-driven cancers provides the opportunity to specifically stimulate and expand tumor-specific T cells for ACT. Stimulation of peripheral blood lymphocytes with overlapping peptide pools resulted in the expansion of HPV16-specific T cells in 33 of 52 HPV16+ OPSCC patients. Most of the cell cultures comprised HPV16-specific CD4+ T cells and infrequently CD8+ T cells with the capacity to kill target tumor cells (122). A highly reproducible method to stimulate these cells under good manufacturing practice (GMP)-conditions resulted in the expansion of IFNγ-producing HPV-specific CD4+ T cells (11 of 11 cases) and CD8+ T cells (3 of 11) cases (131). In a phase II ACT trial, one out of 5 OPSCC patients resolved most of the metastatic lesions (complete response, CR) except for one brain metastasis, which was resected. Responding patients received higher numbers of IFNγ-producing HPV-specific TILs and the frequency of HPV-specific T cells in the blood one month after ACT correlated with clinical outcome. The question is how HPV-specific ACT will be used as most patients of whom their primary tumors display HPV-specific TIL reactivity respond well to chemoradiation while the tumors of most patients with recurrent disease are expected to contain no or few HPV-specific TILs (6). If present in the tumor, ex-vivo expansion and reinfusion of HPV-specific TIL may help to prevent or to reduce chemoradiation as primary treatment.

The absence of HPV-specific T cells mandates alternative approaches such as the infusion of ex-vivo TCR-transduced autologous T cells. Draper *et al.* reported the isolation of an HLA-A*0201 restricted HPV16 E6_29-38_-specific TCR from a patient with HPV16-induced anal cancer. The use of this TCR is expected to contribute to tumor immunity as the tumor site was highly enriched for this TCR clonotype when compared to blood and T cells transduced with this TCR were shown to recognize several HPV16+ cell lines of different origins (159). Another study reported the isolation of an HLA-DRB1*04 restricted TCR reactive to the HPV16 E7_70-89_ epitope. TCR-transduced cells were shown to specifically produce IFNγ when stimulated with HPV16 E6 and E7 containing tumor lysate (160). The isolation of TCR responsive to HPV epitopes restricted by a series of HLA class I and II molecules will lead to a warehouse of HPV-specific TCR required for personalized treatment of patients with recurrent or metastatic HPV-induced OPSCC. Currently, the use of T cells transgenic for an E7-specific TCR is tested in the clinic (**Table 1**). The fact that most mutations (neoantigens) are patient specific makes a similar approach for HPV-negative OPSCC complicated but not impossible (161).

### Vaccines

Therapeutic vaccination is another option to increase the number of tumor-reactive T cells in OPSCC, preferentially against tumor-specific epitopes created by DNA mutations or oncogenic viruses. Based on a whole series of different trials it was concluded that the induction of tumor reactivity correlated with clinical outcome after vaccination in patients with pre-cancers or in settings of low tumor burden, and with tumor regressions if broad type 1 T cell reactivity was established (162). The treatment of most established cancers will require the combination of therapeutic vaccination with other modalities to overcome immune suppression and escape (163).

Two types of vaccines, one aiming to induce responses to MAGE-A3 and HPV and the other aiming to induce T-cell reactivity against p53 (for HPV-negative tumors) were tested in groups of HNSCC patients, including 30–40% OPSCC patients. These vaccines displayed a weak immunogenicity and did not alter clinical outcome (164, 165).

However, in a single arm phase II trial, designed based on the observation that HPV16+ OPSCC patients lacking tumor infiltration with HPV16-specific T cells are most likely the ones that are diagnosed with incurable HPV16+ cancer (6, 24) and that PD-1 checkpoint therapy has most impact in patients with pre-existing tumor-specific T cell responses (155), HPV16+ OPSCC patients received a combined treatment with ISA101 and nivolumab (146). ISA101 is an HPV16 -E6 and -E7-specific synthetic long peptide vaccine, which previously was shown to induce CR and PR in patients with HPV16-induced high-grade but premalignant disease (166, 167). The ORR of 33% and median OS of 17.5 months was regarded promising (146) when compared to PD-1 inhibition only in a similar patient group (143) or when compared to the study with durvalumab in which none of the HPV+ patients responded (145). A randomized trial comparing the efficacy of PD-1 blockade alone versus vaccination + PD-1 blockade is underway (**Table 1**). The outcome of the combination ISA101 and nivolumab trial argue for tumor-specific vaccination strategies. Approaches to stimulate the neoantigen repertoire are also warranted for the treatment of in particular HPV-negative OPSCC, similar to what has been done in other types of cancers (168–170).

Another option to stimulate tumor-reactive T cells might be through irradiation, as this has been shown to result in the release of antigens from tumor cells as well as in the induction of proinflammatory signals that trigger the innate immune system (171). Upon radiation, the tumor may serve as an *in situ* vaccine with the capacity to activate tumor-specific T cells. Spurred by the results with checkpoint blockade after radiation therapy in lung cancer (172), new trials are initiated in patients with HNSCC, including OPSCC (**Table 1**), using radiotherapy and checkpoint inhibition together or in combination with chemotherapy (173–175). The feasibility of using PD-L1 antibody avelumab with conventional cetuximab-radiotherapy as an alternative for advanced stage OPSCC patients unfit for cisplatin treatment was tested in a phase I study with 8 patients with OPSCC, 4 of which were HPV+. Immune toxicity was transient and manageable, and objective responses were observed in 4 of 6 evaluable OPSCC patients (3 complete responses and one partial response), including 3 HPV+ patients (176).

## Preclinical Developments

There are several mouse models for HPV+ HNSCC. Study of cell surface proteins expressed by MTEC tumors, made from mouse tonsillar epithelial cells that are transformed by HPV16 E6, E7, and hRAS, showed the expression of CD47 (177). By binding to signal-regulatory protein alpha (SIRP-alpha) expressed on antigen processing cells, this transmembrane molecule blocks the phagocytosis and clearance of cells expressing CD47, resulting in the suppression of innate and adaptive anti-tumor responses. Targeting of CD47 using antibodies in rituximab-refractory non-Hodgkin’s lymphoma re-sensitized about 50% of these patients (178). The CD47-SIRP-alpha axis can also be targeted pharmacologically (179). The efficacy of radiation, which is a key component of the standard of care treatment for OPSCC, was shown to depend on intact T cell responses and was improved after CD47 knock-down in MTEC tumors (177), suggesting that CD47 may also play a key role in regulating immunity and as such the efficacy of standard of care treatment in OPSCC. Another study with mEER tumor cells, derived from the metastases of an HPV+ oropharyngeal murine cancer, injected into the flank of mice showed that the response to standard cisplatin-radiation therapy could be improved by adding cyclophosphamide and an inducible nitric oxide synthase (iNOS) inhibitor. Standard therapy did not alter the tumor microenvironment, which remained cold as indicated by high numbers of immune suppressive immune cells (MDSC, Tregs) and low numbers of T cells, M1 macrophages, and DCs (180). The addition of cyclophosphamide to the standard treatment converted treated tumors to hot but the combination with the iNOS inhibitor resulted in a strong influx of CD8+ and CD4+ Th1 cells as well as lead to durable control of established tumors (180). The use of cisplatin chemotherapy has been shown to increase tumor immune infiltration (181) and tumor cell killing by tumor necrosis factor alpha (TNFα)-producing T cells (182). The use of a low dose cisplatin was also shown to augment the effects of an adenovirus-based oncolytic virus therapy of subcutaneously injected mEER tumors. The effects of the oncolytic virus were clearly CD8+ T cell dependent and cisplatin was shown to augment the infiltration of the mEER tumors with HPV16 E7-specific CD8+ T cells (183). Interestingly, the anatomical location of the mEER tumors had a profound impact on the composition of the tumor infiltrating immune cells (184), quite reminiscent of what was observed in patients with HPV16-induced OPSCC and cervical cancer (24). Orthotopic injection of mEER tumor cells resulted in a much more inflamed tumor immune microenvironment, reflected by higher numbers of tumor-infiltrating CD8+ T cells and a stronger type 1 and 2 gene signature, than when these tumor cells were injected in the flank. Moreover, whereas the orthotopic growing mEER tumors were directly responsive to treatment with anti-PD-1 (> 50% survival) and even better to the combination of anti-PD-1 and anti-CTLA-4 (> 90% survival), flank injected tumors did not show any response to PD-1 blockade alone whereas only 40% responded to the combination treatment. Intratumoral injections of STING agonist, to increase IFN signaling, resulted in a strong decrease of Tregs and MDSC and an almost complete eradication of flank tumors (184). The decrease of immune suppressive immune cells in flank-injected mEER tumors seems essential for a better outcome after both standard chemoradiation (180) and immunotherapy (184).

## Perspectives

The use of 15 parameter flow cytometry, the development of over 30 marker panels by mass cytometry and the introduction of unbiased bioinformatical approaches to cluster cells based on the expression of all proteins assessed have led to a much better definition and quantification of the immune cell phenotypes that are present in OPSCC, solved debates on the prognostic role of certain subtypes and led to the identification of subtypes strongly associated with clinical outcome. The use of single cell RNA sequencing to study the transcriptome of intratumoral cells has provided new means to identify subtypes of immune cells and understand their functional properties as well as defined gene expression signatures with clinical outcome. In a first study of 26 patients with all types of HNSCC, including 7 OPSCC, the transcriptional signatures in helper CD4+ T cells and B cells were quite divergent between HPV-negative and HPV+ tumors, whereas that of CD8+ T cells and Tregs was quite similar (185). These results may not necessarily reflect a difference caused by HPV but can be caused by the different types of tumor and their location (6, 24, 184). In a similar, but purely OPSCC and T cell focused transcriptome study, highly active tumor resident and tumor-reactive populations of CD4+ and CD8+ T cells that displayed actionable checkpoints were found in OPSCC (45). Whilst these techniques give an unprecedented insight in the complexity of the immune infiltrate of tumors, they lack the spatial information on each cell. The fact that a fairly simple classification based on the distribution of T cells has a major impact on prognosis and response to immunotherapy stresses the importance of such analyses (27). Multispectral imaging using the Vectra not only allows for a more in-depth analyses of different types of immune cells within the same tumor section but also to study their interaction. The use of mass cytometry based imaging allows for many more markers to be studied in a spatial context (186) and one can foresee that this will bring a much deeper understanding on the types of cells in the TME as well as their interaction and how this affect clinical outcome.

The much better outcome of HPV+ OPSCC compared to their HPV-negative counterparts after standard of care therapy prompted discussions on de-intensified treatments, specifically with respect to the dose of cisplatin chemotherapy (15), but this led to inferior survival (17). It should be realized that the reason why patients with HPV+ tumors do better on this standard therapy is because of their extensive immune infiltration and this is enhanced by cisplatin (67, 180). Thus, rather than downscaling the use of chemotherapy one should consider to make optimal use of it. For instance, by taking advantage of the fact that chemotherapy may remove some of the immunosuppressive mechanisms that are at play in HPV-induced cancers (187, 188). Rational combination of the immunomodulatory properties of chemotherapy and radiation that turns up the heat in tumors are highly warranted. Based on a recent publication on therapeutic vaccination (189), it is unlikely that checkpoint therapy or ACT approaches will manage to do this. Potentially, oncolytic virus therapy may aid in this (NCT02626000).

Finally, checkpoint therapy has only limited effect in patients with head and neck cancer. One of the problems may be that these patients failed to or mounted only a weak response to tumor antigens. Whereas this can be achieved with therapeutic vaccines in patients with HPV+ tumors, this is more difficult when the tumor antigens are unknown, as is the case in HPV-negative OPSCC. To increase the number of tumor-reactive T cells, approaches are undertaken to either transfer TCR transgenic T cells into patients or by isolation and expansion of T cells which are most likely to be enriched for tumor-reactive T cells using the expression of the activation markers CD137, PD-1, or CD39 to select the T cells (190–193). These approaches have mostly targeted tumor-reactive CD8+ T cells, but in view of the important role (194–196) and efficacy of tumor-reactive CD4+ T cells (197–199) strategies to rapidly isolate that specific T cell fraction are needed. This may be achieved using the expression of CD39 on tumor-infiltrating CD4+ T cells (45).

## Author Contributions

The authors contributed equally to the review. All authors contributed to the article and approved the submitted version.

## Funding

This study was financially supported by grants from the Dutch Cancer Society 2016–10726 to SB, MW, and SS.

## Conflict of Interest

The authors declare that the research was conducted in the absence of any commercial or financial relationships that could be construed as a potential conflict of interest.
